# Effects of Soil Organic Matter Properties and Microbial Community Composition on Enzyme Activities in Cryoturbated Arctic Soils

**DOI:** 10.1371/journal.pone.0094076

**Published:** 2014-04-04

**Authors:** Jörg Schnecker, Birgit Wild, Florian Hofhansl, Ricardo J. Eloy Alves, Jiří Bárta, Petr Čapek, Lucia Fuchslueger, Norman Gentsch, Antje Gittel, Georg Guggenberger, Angelika Hofer, Sandra Kienzl, Anna Knoltsch, Nikolay Lashchinskiy, Robert Mikutta, Hana Šantrůčková, Olga Shibistova, Mounir Takriti, Tim Urich, Georg Weltin, Andreas Richter

**Affiliations:** 1 University of Vienna, Department of Microbiology and Ecosystem Science, Division of Terrestrial Ecosystem Research, Vienna, Austria; 2 Austrian Polar Research Institute, Vienna, Austria; 3 University of Vienna, Department of Ecogenomics and Systems Biology, Division of Archaea Biology and Ecogenomics, Vienna, Austria; 4 University of South Bohemia, Department of Ecosystems Biology, České Budějovice, Czech Republic; 5 Leibniz Universität Hannover, Institut für Bodenkunde, Hannover, Germany; 6 University of Bergen, Centre for Geobiology, Department of Biology, Bergen, Norway; 7 Central Siberian Botanical Garden, Siberian Branch of Russian Academy of Sciences, Novosibirsk, Russia; 8 VN Sukachev Institute of Forest, Siberian Branch of Russian Academy of Sciences, Krasnoyarsk, Russia; 9 International Atomic Energy Agency, Joint FAO/IAEA Division for Nuclear Techniques in Food and Agriculture, Soil and Water Management & Crop Nutrition Laboratory, Vienna, Austria; University of Tartu, Estonia

## Abstract

Enzyme-mediated decomposition of soil organic matter (SOM) is controlled, amongst other factors, by organic matter properties and by the microbial decomposer community present. Since microbial community composition and SOM properties are often interrelated and both change with soil depth, the drivers of enzymatic decomposition are hard to dissect. We investigated soils from three regions in the Siberian Arctic, where carbon rich topsoil material has been incorporated into the subsoil (cryoturbation). We took advantage of this subduction to test if SOM properties shape microbial community composition, and to identify controls of both on enzyme activities. We found that microbial community composition (estimated by phospholipid fatty acid analysis), was similar in cryoturbated material and in surrounding subsoil, although carbon and nitrogen contents were similar in cryoturbated material and topsoils. This suggests that the microbial community in cryoturbated material was not well adapted to SOM properties. We also measured three potential enzyme activities (cellobiohydrolase, leucine-amino-peptidase and phenoloxidase) and used structural equation models (SEMs) to identify direct and indirect drivers of the three enzyme activities. The models included microbial community composition, carbon and nitrogen contents, clay content, water content, and pH. Models for regular horizons, excluding cryoturbated material, showed that all enzyme activities were mainly controlled by carbon or nitrogen. Microbial community composition had no effect. In contrast, models for cryoturbated material showed that enzyme activities were also related to microbial community composition. The additional control of microbial community composition could have restrained enzyme activities and furthermore decomposition in general. The functional decoupling of SOM properties and microbial community composition might thus be one of the reasons for low decomposition rates and the persistence of 400 Gt carbon stored in cryoturbated material.

## Introduction

Decomposition of soil organic matter (SOM) depends on extracellular enzymes, produced by microorganisms. Microbes exude enzymes to acquire carbon (C) or limiting nutrients [1], and to target the most abundant substrates [2]. Extracellular enzyme activities are therefore often related to the chemical composition of SOM and its C and nitrogen (N) content [3], [4].

SOM quality and quantity are, however, not the only controls on microbial enzyme activities. Extracellular enzymes have often been found to be related to microbial diversity or the abundance of individual microbial groups [5], [6]. Microbial community composition in turn is shaped by environmental factors such as temperature, moisture, O_2_ availability and pH [7]. Changes in these factors can promote specific microbial groups that are better adapted to the new environment, but these microbial groups might also differ in their functional properties. This could in turn alter microbial enzyme activities, microbial processes and ultimately decomposition of SOM [8], [9].

In most soils, edaphic factors including pH, nutrient levels, and the quantity and quality of SOM change with depth from horizon to horizon [7], [10]. Along this gradient also microbial biomass decreases [11]–[13], and microbial community composition shifts [7], [14], [15]. These co-correlations and interactions of different factors make it difficult to identify the main drivers of microbial community composition and microbial processes. Attempts to dissect the possible drivers in manipulative experiments have shown that changes of environmental conditions and the inoculation of a specific microbial community onto a non-native substrate can both cause constrained microbial functions [16], [17].

One example where SOM is subjected to unfamiliar abiotic environmental conditions can be found in arctic soils. In the arctic permafrost region, freeze-thaw processes cause the subduction of C-rich topsoil material into deeper soil layers [18]. This cryoturbated material is poorly decomposed and similar to topsoil horizons in terms of SOM composition and C and N content [19]–[21]. Cryoturbated permafrost soils are wide spread in the Arctic and cryoturbated material accounts for up to 400 Gt of carbon [22]. The main reason for the persistence of these large amounts of C is most likely connected to the position of cryoturbated material deep in the soil profile, where abiotic environmental conditions are quite different from those in topsoil horizons. Lower temperatures and O_2_ contents, higher water contents, and shorter frost-free periods are related to soil depth [23], [24], and are therefore similar in cryoturbated material and in surrounding mineral subsoil horizons. In a previous study, we found that microbial community composition was similar in cryoturbated material and mineral subsoil horizons and different in topsoil horizons, although C and N contents of cryoturbated material were similar to mineral topsoil horizons [25]. The decoupling of SOM properties and microbial community composition could influence the production of extracellular enzyme and thus impair the ability of the microbial community to decompose organic matter.

The objective of this study was to identify the effect of soil properties (C and N content) and microbial community composition on microbial enzyme activities in cryoturbated arctic soils. We constructed structural equation models for three potential enzyme activities: the C-acquiring enzyme cellobiohydrolase (CBH), the N-acquiring enzyme leucine-amino-peptidase (LAP) and the oxidative enzyme phenoloxidase (POX), which is involved in the decomposition of complex organic substances [3]. We also included C and N content, clay content, water content, pH, and microbial biomass, as well as microbial community composition derived from phospholipid fatty acid (PLFA) analysis as potential drivers in the models. All data were obtained from measurements of soil samplesfrom three regions in the Siberian Arctic: Northeast Siberia (Kolyma area), Central Siberia (Taymyr peninsula) and Western Siberia (near Tazovsky). Samples were taken from main horizons and pockets of cryoturbated material. We hypothesized that over all three arctic sites microbial community composition would be similar in cryoturbated material and in the surrounding subsoil. Because of the decoupling of SOM properties (similar in topsoil and cryoturbated material) and microbial community composition in cryoturbated material, we expected the microbial community composition to stronger influence microbial enzyme activities in cryoturbated material than in regular soil horizons.

## Material and Methods

### Sites and sampling

**Figure 1:**
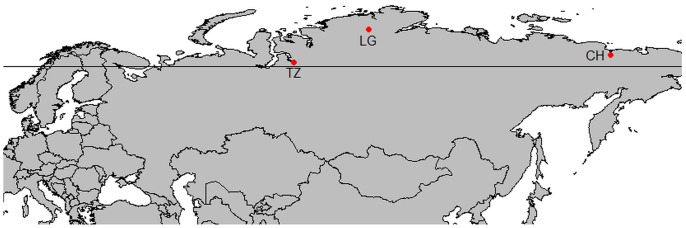
Map showing the three sampling sites in the Siberian Arctic. Tazovsky (TZ; 67°16′N, 78°50′E), Logata (LG; 73°25′N, 98°16′E) and Cherskiy (CH; 68°45′N, 161°20′E). The horizontal line is the polar circle.

We collected soil samples from three regions in the Siberian Arctic (Figure 1). Sampling was carried out in northeast Siberia near Cherskiy (68°45′N, 161°20′E), around 180 km north-west of the town Khatanga, at the river Logata (73°25′N, 98°16′E), and near the town of Tazovsky (67°16′N, 78°50′E). Basic climate data and vegetation classes for the three sites can be found in Table 1. Vegetation classes were determined in the field. All climate data are interpolated data of records from 1950–2000 derived from WorldClim [26]. No specific permissions were required for these locations and activities. Our study did not involve endangered or protected species.

**Table 1:** Climate and Vegetation.

	Coordinates	Year of sampling	Vegetation classes	MAT	Tmax	Tmin	MART	MAP
				°C	°C	°C	°C	mm
Cherskiy (CH)	68°45′N, 161°20′E	2010	shrubby grass tundra	−12.7	14.0	−35.9	49.9	160
			shrubby tussock tundra					
			shrubby lichens tundra					
Logata (LG)	73°25′N, 98°16′E	2011	dryas tundra	−13.5	14.4	−36.6	51.0	270
			grassy moss tundra					
Tazovsky (TZ)	67°16′N, 78°50′E	2012	shrubby lichens tundra	−8.2	17.2	−29.9	47.1	454
			larch woodland with shrubby lichens understory					

Climate data are derived from WorldClim database including mean annual temperature (MAT), maximum temperature of the warmest month (Tmax), minimum temperature of the coldest month (Tmin) mean annual range in temperature (MART) and mean annual precipitation (MAP).

In each region, we chose two or three land cover types in which we sampled three 5 m-long soil pits each, down to the permafrost. Soil samples were taken from all distinct horizons of the active layer, including pockets of cryoturbated topsoil material in the mineral subsoil.

Since all of the horizons were affected by cryoturbation processes and varied in size, we accounted for spatial variability in the field by pooling subsamples from several places within each of the horizons of a single pit. We sampled between 300 g and 500 g of fresh soil per horizon.

To avoid any influence of cut-off roots on enzyme activities and phospholipid fatty acids (PLFAs), living roots were carefully removed before further analyses [27]. Soil horizons were grouped in organic topsoil (n = 17), mineral topsoil (n = 18), mineral subsoil (n = 23), and cryoturbated material (n = 43). Organic topsoils included horizons with an organic C content of more than 17% [28]. Mineral topsoils contained topsoil horizons including O/A-horizons and A-horizons with C content below 17%. B, C and BC horizons were combined in the category mineral subsoils. Although all horizons in these arctic soils can be turbated, horizons in the categories organic topsoil, mineral topsoil and mineral subsoil roughly followed a depth-related decrease in C content, and will further on be referred to as regular soil. In contrast to regular soil, subducted, SOM-rich material, which was surrounded by mineral subsoil material with a significantly lower C content, will be further on referred to as cryoturbated material.

### Soil properties

Clay content was determined using a pipette method following DIN 66100 (German Industrial Standard). Water content was determined gravimetrically by drying and expressed as percent of fresh mass. Soil pH was determined in a water suspension at a solid to solution ratio of 1:2.5. After acid fumigation for 48 h to remove carbonates [29], samples were analyzed for total organic carbon (C) and nitrogen (N) contents using an EA-IRMS system (Vario ISOTOPE cube, Elementar, Hanau, Germany).

### Potential extracellular enzyme activities

We measured potential enzyme activities fluorimetrically and photometrically using a microplate assay [6]. For the fluorimetric assay we used 4-methylumbelliferyl-β-D-cellobioside, and L-leucine-7-amido-4-methyl coumarin as substrates for cellobiohydrolase (CBH) and leucine-amino-peptidase (LAP), respectively. Phenoloxidase (POX) activities were measured using L-3,4-dihydroxiphenylalanine (DOPA) as substrate in a photometric assay. Assays for CBH and LAP were incubated for 140 min at room temperature in a sodium-acetate-buffer (pH 5.5) and measured afterwards fluorimetrically (excitation 365 nm and emission 450 nm). POX assay was measured photometrically (absorbance 450 nm) immediately and after incubation for 20 hours at room temperature.

### Phospholipid fatty acid (PLFA) analysis

Samples for analysis of PLFA were stored in RNAlater prior to extraction [30]. Extraction and measurement followed the procedure described by Frostegård et al. [31] with the modifications described by Kaiser et al. [6]. In short, PLFAs were extracted from 1 g of soil with chloroform/methanol/citric acid buffer and purified on silica columns (LC-Si SPE, Supleco, Bellefonte, PA, USA) using chloroform, acetone, and methanol. After addition of the internal standard (methyl-nonadecanoate), PLFAs were converted to fatty acid methyl esters (FAMEs) by alkaline methanolysis. Samples were analyzed on a Thermo Trace GC with FID detection (Thermo Fisher Scientific, Waltham, MA, USA), using a DB-23 column (Agilent, Vienna, Austria). For quantification of the marker 10Me16:0 we additionally used a DB-5 column (Agilent, Vienna, Austria). FAMEs were identified using qualitative standard mixes (37 Components FAME Mix and Bacterial Acid Methyl Esters Mix, Supelco) and quantified using the internal standard. We categorized the fatty acids 16:1ω5, 18:1ω9, 18:2ω6,9, and 18:3ω3,6,9 as markers for fungi; i15:0, a15:0, i16:0, i17:0, and a17:0 as markers for gram positive bacteria; cy17:0 (9/10), cy18:0 (11/12), cy19:0 (9/10), 16:1ω7, 16:1ω9, and 18:1ω7 for gram negative bacteria; 10Me16:0 as marker for actinobacteria; 15:0, 17:0, 17:1ω6, 18:1ω5 as general bacterial markers. All markers for gram positive bacteria, gram negative bacteria, actinobacteria and general bacteria markers were used to calculate bacterial biomass and fungi: bacteria ratios. We used the above mentioned markers together with 14:0, i14:0, 16:0, 18:0, 20:0, 16:1ω11, and 19:1ω8 as a proxy for microbial biomass [6].

### Statistics

Before statistical analyses, data were checked for normality, and log-transformed or rank-normalized if necessary. To determine if differences in enzyme activities and microbial community composition were greater between horizons or between sites, we performed two-way-ANOVAs. Differences in soil parameters and enzyme activities between horizons were addressed by ANOVA and Tukey-HSD. To find differences in microbial community composition, we performed principal component analysis (PCA) with the relative abundances of all PLFA markers from all samples, and analyzed the PC-axes with ANOVA and Tukey-HSD test afterwards. When p-values were below 0.05, differences were considered significant.

To find differences in the causal associations in our data, we used structural equation modeling (SEM) following Colman and Schimel [32] and Grace [33]. We constructed a conceptual base model using clay content, water content, pH, C and N contents, microbial biomass, and microbial community composition (PC1, PC2, PC3 from the PLFA-PCA) as direct and indirect factors controlling individual enzyme activities. Clay content in O-horizons was assumed to be zero. The models were run for regular soil (organic topsoil, mineral topsoil and mineral subsoil) and cryoturbated material separately, using PCA axes from separate PCAs run individually for the two groups. Climate parameters were not included in the models, since these parameters represent topsoil conditions and might therefore bias models describing differences between horizons. To achieve a representative model, indicated by a low model chi-squared (χ^2^) and a high model p-value (P>0.05), individual paths or variables were removed or added. Model optimization was done by removing not significant paths (P>0.05). Alternative models were compared using Akaike's Information Criterion (AIC) to determine the most parsimonious model. All statistical analyses, calculations and maps were performed and created in R version 3.0.0 [34].

## Results

### Soil properties

In regular soil, both C and N content, and C:N ratios decreased from organic topsoil over mineral topsoil to mineral subsoil (Table 2). In cryoturbated material these parameters were in the range of mineral topsoil, and were always significantly different from organic topsoil and mineral subsoil (Table 2). Water content was highest in organic topsoils followed by mineral topsoil and cryoturbated material and mineral subsoil. pH was significantly higher in subsoil mineral horizons and cryoturbated material than in organic topsoil and mineral topsoil horizons. Interestingly, clay content was highest in cryoturbated material (27.7%) followed by mineral topsoil (22.1%) and mineral subsoil (21.9%) (Table 2).

**Table 2:** Soil properties of the different horizon categories.

	Samplin depth	Organic carbon (C)	Total nitrogen (N)	C:N ratio	Water content	pH	Clay
	cm	%	%	w/w	% of fresh soil		%
**Organic topsoil (n = 17)**		**25.2±1.29 (a)**	**0.98±0.06 (a)**	**26.7±1.77 (a)**	**65.5±2.01 (a)**	**5.16±0.11 (a)**	**n.a.**
Cherskiy (n = 9)	0–30	25.5±1.72	1.12±0.06	23.0±1.48	64.9±2.98	5.13±0.08	n.a.
Logata (n = 3)	0–30	25.4±3.39	1.01±0.06	25.1±2.78	70.1±5.19	5.61±0.21	n.a.
Tazovsky (n = 5)	0–20	24.5±2.06	0.72±0.02	34.2±2.73	64.0±2.25	4.97±0.25	n.a.
**Mineral topsoil (n = 18)**		**8.53±1.33 (b)**	**0.45±0.06 (b)**	**18.0±0.79 (b)**	**40.8±3.49 (b)**	**5.50±0.13 (a)**	**22.1±2.38 (ab)**
Cherskiy (n = 2)	0–40	9.69±4.10	0.43±0.16	21.5±1.73	50.5±14.7	4.80±0.33	7.77±5.50
Logata (n = 11)	0–30	11.0±1.37	0.58±0.06	18.5±0.97	45.6±3.24	5.69±0.15	12.4±3.97
Tazovsky (n = 5)	0–20	2.67±0.61	0.17±0.03	15.6±0.70	26.4±3.70	5.36±0.16	23.9±2.73
**Mineral subsoil (n = 23)**		**1.10±0.17 (c)**	**0.09±0.01 (c)**	**11.5±0.57 (c)**	**20.7±1.01 (c)**	**6.34±0.11 (b)**	**21.9±1.41 (b)**
Cherskiy (n = 5)	10–90	1.58±0.32	0.13±0.02	11.9±0.84	17.6±0.89	5.81±0.10	18.3±1.04
Logata (n = 7)	5–60	1.85±0.10	0.14±0.00	13.6±0.43	25.2±0.22	6.46±0.13	29.6±1.71
Tazovsky (n = 11)	5–100	0.40±0.06	0.04±0.00	10.0±0.80	19.2±1.59	6.50±0.17	18.5±1.38
**Cryoturbated material (n = 43)**		**8.39±0.74 (b)**	**0.48±0.04 (b)**	**16.6±0.56 (b)**	**40.0±1.59 (b)**	**6.06±0.09 (b)**	**27.7±1.28 (a)**
Cherskiy (n = 15)	20–80	9.84±1.30	0.61±0.07	15.7±0.43	40.5±2.33	5.74±0.11	29.5±2.21
Logata (n = 19)	15–50	8.37±0.76	0.47±0.03	17.3±0.88	43.6±1.96	6.45±0.10	23.3±2.20
Tazovsky (n = 9)	20–90	6.00±1.98	0.30±0.08	16.7±1.65	31.6±4.32	5.78±0.22	21.6±4.81

Values are mean values (± standard error) over all sites and for each horizon per site. Letters in parentheses indicate significantly different (P<0.05) groups between horizons derived from ANOVA and Tukey-HSD tests.

### Microbial enzyme activity

**Figure 2:**
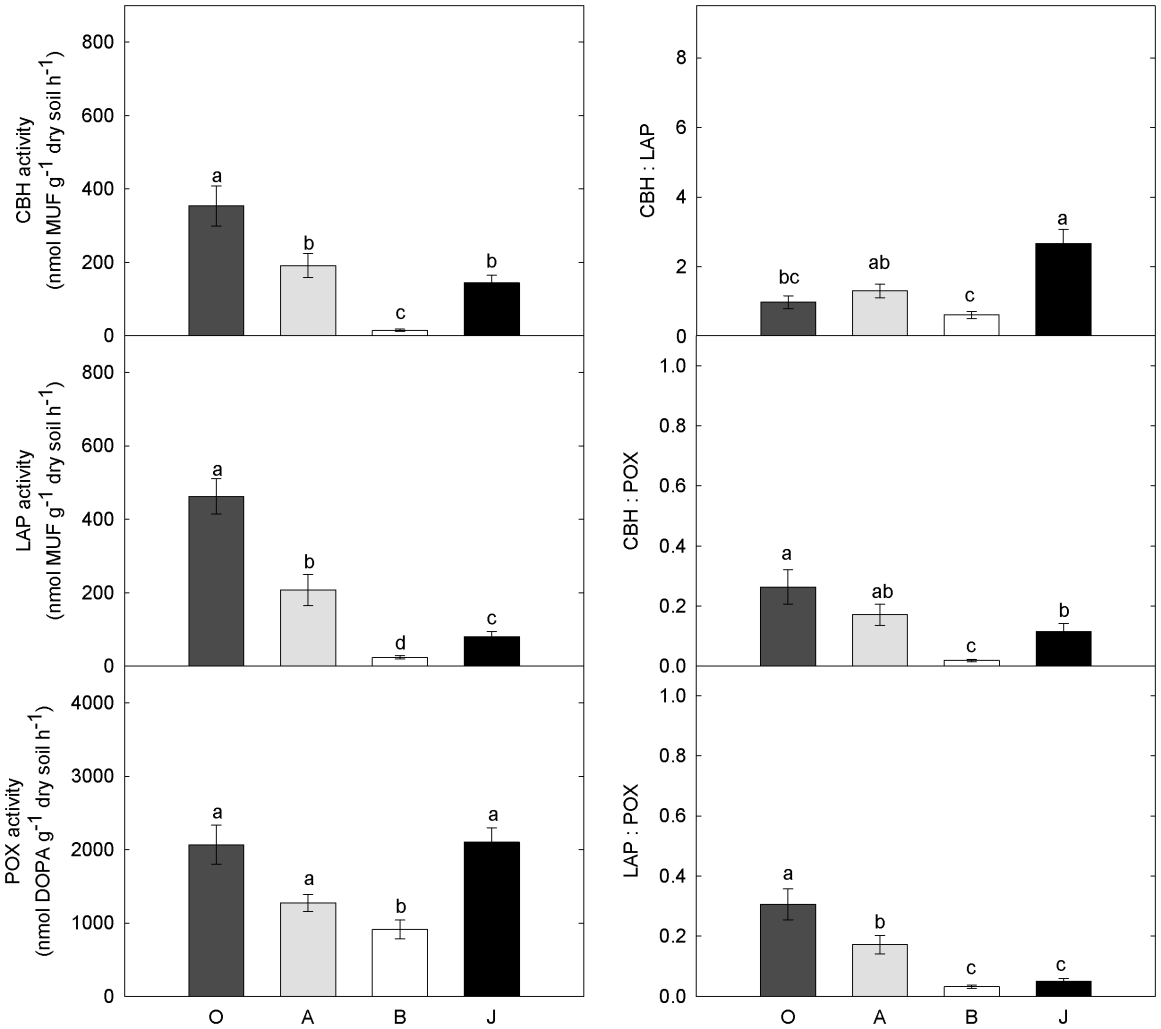
Extracellular enzyme activities and enzyme ratios in different horizons in arctic soils. Left panel: Extracellular enzyme activities for the C-acquiring enzyme cellobiohydrolase (CBH), the N-acquiring enzyme leucine-amino-peptidase (LAP) and the oxidative enzyme phenoloxidase (POX). Right panel: Ratios of the three enzyme activities to each other. Given are the means and standard errors for the individual horizon categories: organic topsoil (O), mineral topsoil (A), mineral subsoil (B), and cryoturbated material (J). Colors indicate different horizon categories: organic topsoil is dark grey, mineral topsoil is light grey, mineral subsoil is white, and cryoturbated material is black. Small letters indicate different statistical groups derived from ANOVA and Tukey-HSD tests.

All enzyme activities were significantly different between horizons and sampling sites, but differences between horizons had the greater explanatory power (Table S1). Hydrolytic enzyme activities (CBH and LAP) decreased from organic topsoil over mineral topsoil to mineral subsoil, showing the same patterns as the decrease of C and N content from organic topsoil over mineral topsoil to mineral subsoil (Figure 2). POX activity was significantly higher in mineral subsoil than in organic and mineral topsoil horizons (Figure 2).

Although cryoturbated material and mineral topsoil had similar C and N contents (Table 2), only CBH and POX activites were similar, whereas LAP activity was significantly lower in cryoturbated material (Figure 2). CBH:LAP ratios, as an indicator of the C:N acquisition activity, were highest in cryoturbated material and mineral topsoil followed by organic topsoil and mineral subsoil. The ratios of CBH:POX and LAP:POX were both significantly lower in cryoturbated material than in organic topsoils (Figure 2), and LAP:POX was even significantly lower in than in mineral topsoils.

### Microbial community

**Figure 3:**
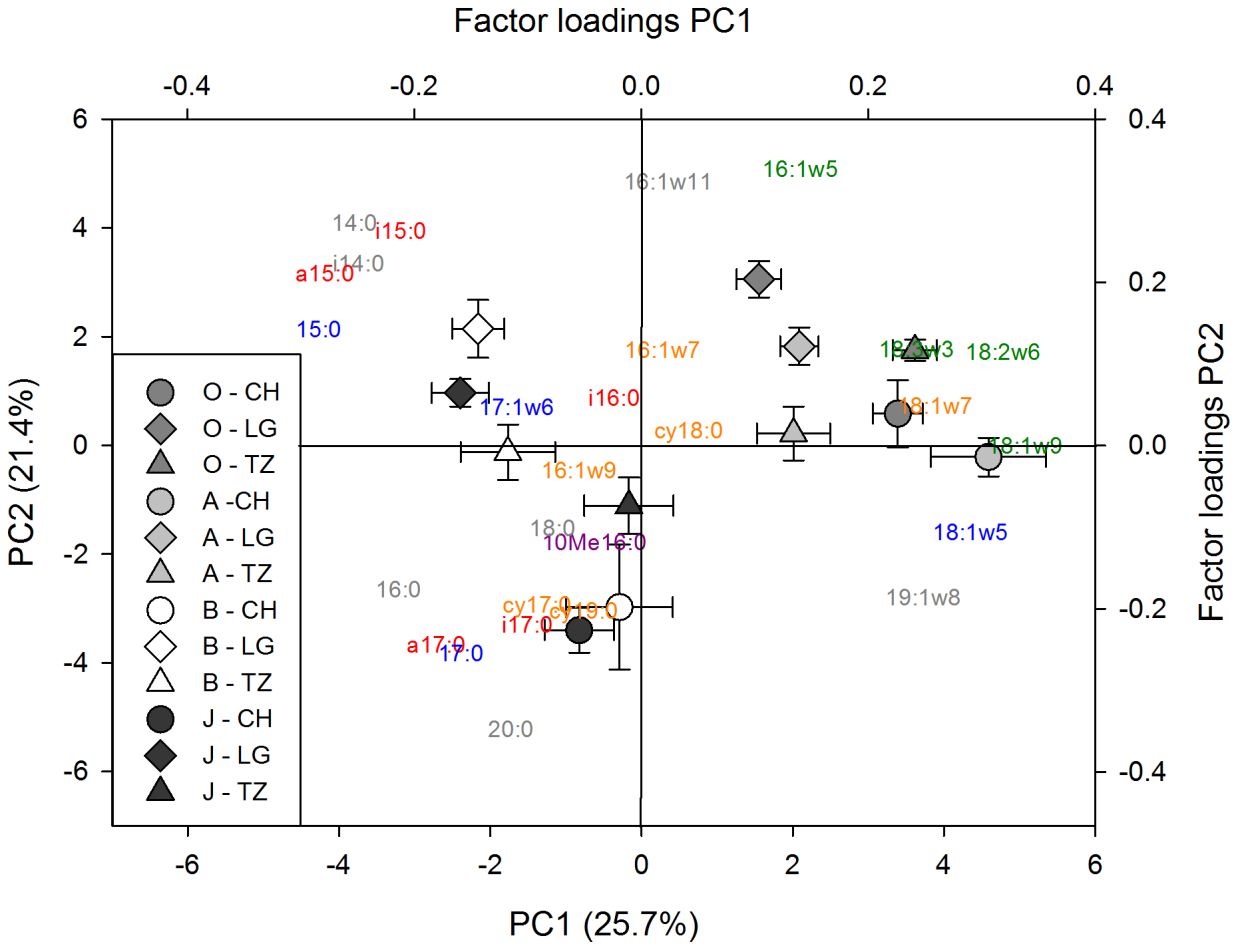
Differences in microbial community composition in different horizons in arctic soils. Principal component analysis (PCA) with relative abundances of all PFLA biomarkers. Colors indicate different horizon categories: organic topsoil (O) is dark grey, mineral topsoil (A) is light grey, mineral subsoil (B) is white, and cryoturbated material (J) is black. Symbols indicate sites: circles Cherskiy, diamonds Logata, and triangles Tazovsky. Symbols are the mean values of the coordinates for the individual categories, derived from the PCA with individual samples (n = 101). Error bars are SE. Colors of PLFA markers indicate general markers (grey), gram-positive markers (red), gram-negative markers (orange), bacterial markers (blue) and fungal markers (green).

To assess microbial community composition, we analyzed PLFAs and used the obtained relative abundances of 27 individual biomarkers from all sites in a PCA. The PCA showed a clear separation of topsoil (organic and mineral) on one side and subsoil mineral and cryoturbated material on the other side along the PC1 axis (Figure 3). The effect of horizon (R^2^ = 0.58) was stronger than that of sampling site (R^2^ = 0.07; Table S1). Along PC2, horizons were similarly separated, but site effects were stronger (R^2^ = 0.43) than horizon effects (R^2^ = 0.17; Table S1). PC3 showed no statistically significant differences between horizon categories but a significant effect of sampling site (Table S1). Fungal biomarkers were strongly represented in PC1, and are most likely responsible for the differences in microbial community composition between topsoil (organic and mineral) and subsoil (mineral subsoil and cryoturbated material). These different fungal abundances are also reflected in the fungi:bacteria ratios, which were two times higher in topsoils than in subsoils (Table 3). The results from the PCA, and the patterns in fungi:bacteria ratios, indicate that the microbial community composition in cryoturbated material is similar to that in mineral subsoil, and significantly different (P<0.05) from the composition of the topsoil microbial community.

**Table 3:** Properties of the microbial community.

	Microbial biomass (total PLFAs)	Fungi:bacteria ratio	PLFA PC1	PLFA PC2	PLFA PC3
	nmol g-1 soil				
**Organic topsoil (n = 17)**	**1833±234 (a)**	**0.55±0.05 (a)**	**a**	**a**	**n.s**
Cherskiy (n = 9)	1117±91.4	0.59±0.08			
Logata (n = 3)	2019±290	0.48±0.09			
Tazovsky (n = 5)	3009±274	0.52±0.03			
**Mineral topsoil (n = 18)**	**650±103 (b)**	**0.53±0.05 (a)**	**a**	**a**	**n.s**
Cherskiy (n = 2)	624±321	0.91±0.18			
Logata (n = 11)	675±116	0.51±0.05			
Tazovsky (n = 5)	606±215	0.40±0.06			
**Mineral subsoil (n = 23)**	**59.3±12.9 (d)**	**0.21±0.01 (b)**	**b**	**ab**	**n.s**
Cherskiy (n = 5)	22.3±6.65	0.24±0.03			
Logata (n = 7)	115±27.8	0.21±0.02			
Tazovsky (n = 11)	40.4±10.1	0.19±0.02			
**Cryoturbated material (n = 43)**	**301±41.3 (c)**	**0.21±0.01 (b)**	**b**	**b**	**n.s**
Cherskiy (n = 15)	175±31.4	0.22±0.01			
Logata (n = 19)	336±48.5	0.19±0.01			
Tazovsky (n = 9)	435±140	0.25±0.04			

Total amount of PLFAs, fungi∶bacteria ratios and statistical results for the first three principal components derived from a PCA with relative abundances of all PLFA biomarkers. Values are mean values (± standard error) over all sites and for each horizon per site. Letters in parentheses indicate significantly different (P<0.05) groups between horizons derived from ANOVA and Tukey-HSD tests.

The sum of all PLFAs, a proxy for microbial biomass, was five times higher in cryoturbated material than in mineral subsoil, but lower by a factor of two compared to mineral topsoils, although C and N contents were similar in mineral topsoils and cryoturbated material (Tables 2 and 3).

### Effects of microbial community composition, carbon and nitrogen contents, and soil parameters on enzyme activity

Since we expected different controls on enzyme activities in regular soil (organic topsoil, mineral topsoil and mineral subsoil) and cryoturbated material, we performed structural equation modeling (SEM) individually for regular soil and cryoturbated material. For the structural equation models we used the first three principal components of PCAs, performed separately for regular soil or for cryoturbated material. A table with the factor loadings is provided in (Table S2).

**Figure 4:**
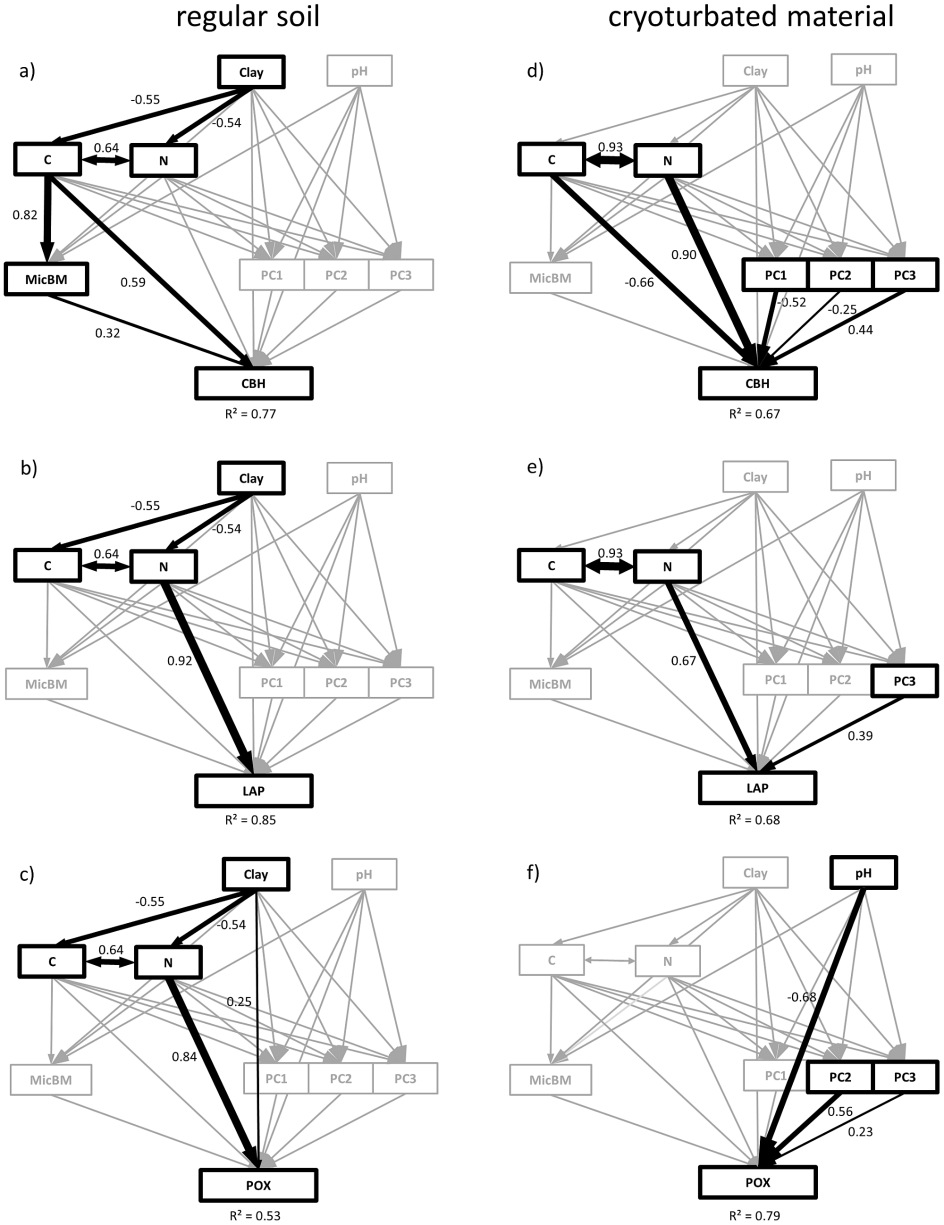
Direct and indirect drivers of extracellular enzyme activities. Structural equation models for extracellular enzyme activities, cellobiohydrolase (CBH; a, d), leucine-amino-peptidase (LAP; b, e) and phenoloxidase (POX; c,f). Graphs on the left show regular soil (organic topsoil, mineral topsoil, mineral subsoil), right panel shows cryoturbated material. Black boxes and arrows indicate significant factors and paths. Boxes and arrows in grey were removed from the model because either the paths (arrows) were not significant, or the factors (boxes) had no direct or indirect effect on the enzyme activity. The boxes with C, N and Clay are the contents of organic carbon, nitrogen, and clay. Microbial biomass (MicBM) was calculated as total amount of PLFAs. PC1, PC2 and PC3 are the first three axes of PCAs with relative abundances of all PLFAs. PCAs for regular soil and cryoturbated material have been done individually. Arrow width indicates the strength of the effect and reflects the scaled estimates, which are also given as the numbers beside the respective arrows. The numbers below the boxes with the respective enzymes show R^2^ and indicate how much of the variance in enzyme activity is explained by the model.

In regular soil, the PCA showed a strong influence of fungal abundance along the first principal component (PC1). The second component (PC2) and the third component (PC3) did not represent specific groups (Table S2). The structural equation models for the three analyzed enzyme activities (CBH, LAP and POX) showed that in regular soils all activities were related to C or N content, but not to microbial community composition (Figure 4). CBH was directly affected by C and microbial biomass with the model explaining 77% of the variance in enzyme activity (R^2^ = 0.77, χ^2^ = 7.04, P = 0.13, Figure 4a). LAP was only directly affected by N (R^2^ = 0.85, χ^2^ = 5.31, P = 0.07, Figure 4b). POX was directly affected by N and clay content (R^2^ = 0.53, χ^2^ = 0.13, P = 0.72, Figure 4c).

In contrast, enzyme activities in cryoturbated material were influenced by microbial community composition. Microbial community composition was represented by principal component axes from a PCA based on PLFA profiles for cryoturbated material only (Table S2). PC1 was not connected to a specific microbial group. PC2, however, reflected a strong influence of fungal versus bacterial markers. Gram positive markers and the actinobacteria marker had the highest negative factor loadings on PC3, whereas gram negative markers had high positive factor loadings.

In contrast to regular soils, the structural equation models for cryoturbated material showed that clay content did not significantly affect C and N content. CBH was affected by C, N and by all three PCA axis representing microbial community composition (R^2^ = 0.67, χ^2^ = 15.29, P = 0.08, Figure 4d), but was not related to microbial biomass. The model for LAP in cryoturbated material explained 68% of the variance in enzyme activity, and showed that only N and PC3-axis significantly affected LAP activity (R^2^ = 0.68, χ^2^ = 4.46, P = 0.22, Figure 4e). POX in cryoturbated material was not affected by C or N, but only by microbial community composition (PC2 and PC3), as well as pH (R^2^ = 0.79, χ^2^ = 1.16, P = 0.76, Figure 4f).

Models including water content were not representative, or had a higher AIC and where therefore dismissed for the models presented in Figure 4.

## Discussion

Despite the differences in SOM quality and quantity, we found similar microbial community composition in cryoturbated material and mineral subsoil. This suggests that the subsoil microbial community, in cryoturbated material, was not fully adapted to its substrate and thus exhibited potentially constraining effects on decomposition by extracellular enzymes.

### Effects of SOM properties on microbial community composition

As opposed to non-cryoturbated soils, where the strong depth dependence of SOM quality and quantity prevents understanding of what influences microbial community composition, the rather unique situation in cryoturbated arctic soils allowed us to elucidate the influence of SOM properties on microbial community composition. Since cryoturbations are up to thousands of years old [21], [36], the microbial community in cryoturbated organic material should by now be in equilibrium with the physical subsoil conditions and topsoil SOM properties. Alternatively one of the two factors could be more important in shaping microbial community composition. We found that the subduction of organic matter, that was qualitatively [21] and quantitatively (Table 2) similar to topsoil mineral horizons, into the mineral subsoil, resulted in a microbial community composition similar to that in mineral subsoil (Figure 2, Table 3). Although PLFA analysis can only resolve microbial community composition at a coarse level, our findings are corroborated by a recent study in North-East Siberia, where molecular techniques were used to determine microbial community composition [25]. In this study, the authors described a strong decrease in fungi:bacteria ratio derived from bacterial and fungal marker genes (16S rRNA gene for bacteria and the ITS for fungi) from topsoil to mineral subsoil and cryoturbated material similar to our findings (Table 3). Taken together, this suggests that the microbial community in cryoturbated material was not well adapted to SOM properties. Similarly, a high-to-low elevation soil translocation in an alpine ecosystem showed only minor effects of substrate quality on microbial community composition assessed by PLFA analysis [37]. In this study, differences in microbial community composition were attributed to different temperatures. Although we did not measure abiotic parameters such as temperature and O_2_ content in situ, these factors might also have shaped the microbial community in cryoturbated material in arctic soils.

### Influence of microbial community composition on microbial enzyme activity

The importance of microbial community composition for microbial processes is currently a hot topic and heavily debated [32], [38]–[40]. For instance, Colman and Schimel [32], argue that microbial community composition has only a minor effect on general microbial processes such as microbial respiration and nitrogen mineralization. In their study of North American continental soils, microbial processes were primarily related to C and N content. In line with these observations, we found no effect of microbial community composition on microbial enzyme activities in regular arctic soil horizons (Figure 4 a–c). Microorganisms in regular soil seem to be in equilibrium with environmental (i.e., edaphic and climatic) conditions and SOM properties, with the result, that although the present microbes produce the enzymes, only substrate properties drive extracellular enzyme activities.

In contrast, in cryoturbated material, the activities of all investigated enzymes were related to microbial community composition (Figure 4 d–f). Here the microorganisms were not adapted to the available substrate, which could have resulted in restrained enzyme activities. This might have caused significantly lower activities of the N-related enzyme LAP in cryoturbated material compared to mineral topsoils (Figure 2). Besides LAP, also the ratio of LAP and POX was up to ten times lower in mineral subsoil and in cryoturbated material compared to topsoil horizons (Figure 2). Although oxidative enzymes, such as POX, are rather unspecific and contribute to the acquisition of both C and N [41], some studies describe oxidative enzymes as predominantly N-acquiring [9]. We found POX to be driven only by N in regular arctic soils, which suggests that POX was at least to a certain extent used to acquire N. Although ratios of hydrolytic enzymes to POX are often interpreted as a measure for recalcitrance of the substrate [35], the ratio between LAP and POX could also be seen as a functional property of the microbial community for N-acquisition. The controlling effect of the microbial community, which seems to be especially pronounced in the degradation of N containing SOM, might also be the cause for a previously described deceleration in the N-cycle in cryoturbated material [42].

The control of the microbial community composition over enzyme activities and decomposition processes might not only be of relevance for decomposition of cryoturbated material in arctic soils. Microbial processes could be controlled similarly in other ecosystems, where the usual equilibrium of SOM and microorganisms might be disrupted, such as in bioturbated soils and in areas, where organic material is repeatedly buried by sedimentation or landslides. Investigation of these systems is warranted to estimate the importance of our findings for C and N cycling on a global level.

## Conclusions

The in situ translocation of topsoil material to the subsoil allowed us to demonstrate that the microbial subsoil community that established in cryoturbated material was not well adapted to SOM properties of cryoturbated material and had a potentially restraining effect on extracellular enzyme activities. The proposed control of microbial community composition might not be limited to potential enzyme activities. Main microbial processes such as C and N mineralization have been found to be strongly reduced in cryoturbated material in arctic soils [6]. This low microbial activity has been held responsible for the high ^14^C age and the low degree of decomposition of this material. A microbial community that is not adapted to the available substrate might be a reason for low microbial activity, and in turn for the current persistence of cryoturbated material in arctic soils.

## Supporting Information

Table S1 **Differences between horizons and between sampling sites.** R^2^ calculated from sum of squares derived from a Two-way-ANOVA for the given parameters and horizon and sampling site as factors. Bold values indicate significant differences (p<0.001). Results for the first three principal components derived from a PCA with relative abundances of all PLFA biomarkers.
(DOCX)

Table S2 **Factor loadings for the first three axes of the Principal Component Analyses used in structural equation models.** PCAs have been performed individually for regular soil, including organic topsoil (O), mineral topsoil (A) and mineral subsoil (B), and for cryoturbated material (J). Markers for individual groups are assigned as following: gram positive bacteria (gram +), gram negative bacteria (gram -), actinobacteria (actino), general bacterial markers (bacteria), fungal markers (fungi) and unspecific markers (general).
(DOCX)
