# Supplementary material for: Effects of Soil Organic Matter Properties and Microbial Community Composition on Enzyme Activities in Cryoturbated Arctic Soils
Source: PLoS One. 2014 Apr 4;9(4):e94076. doi: 10.1371/journal.pone.0094076 (PMC3976392; doi:10.1371/journal.pone.0094076)
Supplement: Table S1 — Differences between horizons and between sampling sites. R2 calculated from sum of squares derived from a Two-way-ANOVA for the given parameters and horizon and sampling site as factors. Bold values indicate significant differences (p<0.001). Results for the first three principal components derived from a PCA with relative abundances of all PLFA biomarkers. (DOCX) [file pone.0094076.s001.docx]

Table S1 Differences between horizons and between sampling sites

|  | Horizon R² | Site R² |
| --- | --- | --- |
| Cellobiohydrolase activity | **0.51** | **0.20** |
| Leucine-amino-peptidase activity | **0.63** | **0.21** |
| Phenoloxidase activity | **0.28** | **0.18** |
| PLFA PC1 | **0.58** | **0.07** |
| PLFA PC2 | **0.17** | **0.43** |
| PLFA PC3 | 0.05 | **0.26** |

R² calculated from sum of squares derived from a Two-way-ANOVA for the given parameters and horizon and sampling site as factors. Bold values indicate significant differences (p<0.001). Results for the first three principal components derived from a PCA with relative abundances of all PLFA biomarkers.
