# Supplementary material for: Effects of Soil Organic Matter Properties and Microbial Community Composition on Enzyme Activities in Cryoturbated Arctic Soils
Source: PLoS One. 2014 Apr 4;9(4):e94076. doi: 10.1371/journal.pone.0094076 (PMC3976392; doi:10.1371/journal.pone.0094076)
Supplement: Table S2 — Factor loadings for the first three axes of the Principal Component Analyses used in structural equation models. PCAs have been performed individually for regular soil, including organic topsoil (O), mineral topsoil (A) and mineral subsoil (B), and for cryoturbated material (J). Markers for individual groups are assigned as following: gram positive bacteria (gram +), gram negative bacteria (gram -), actinobacteria (actino), general bacterial markers (bacteria), fungal markers (fungi) and unspecific markers (general). (DOCX) [file pone.0094076.s002.docx]

| PLFA | Marker | Regular soil | | |  | Cryoturbated | | |
| --- | --- | --- | --- | --- | --- | --- | --- | --- |
|  |  | PC1 (26.3%) | PC2 (18.3%) | PC3 (14.5%) |  | PC1 (30.6%) | PC2 (18.5%) | PC3 (11.1%) |
| i15:0 | gram + | 0.281 | -0.258 | -0.020 |  | -0.284 | 0.010 | -0.217 |
| a15:0 | gram + | 0.315 | -0.193 | -0.067 |  | -0.326 | 0.091 | 0.027 |
| i16:0 | gram + | -0.048 | -0.197 | -0.078 |  | -0.078 | 0.074 | -0.289 |
| i17:0 | gram + | 0.147 | 0.049 | -0.397 |  | 0.219 | -0.028 | -0.353 |
| a17:0 | gram + | 0.207 | 0.152 | -0.260 |  | 0.200 | 0.164 | -0.250 |
| 16:1w9 | gram - | 0.096 | -0.208 | -0.203 |  | 0.055 | 0.260 | 0.252 |
| 16:1w7 | gram - | -0.019 | -0.118 | 0.024 |  | -0.107 | -0.128 | 0.136 |
| cy18:0 | gram - | 0.017 | -0.260 | -0.248 |  | 0.030 | -0.019 | 0.273 |
| cy17:0 | gram - | 0.062 | -0.081 | -0.428 |  | 0.158 | 0.180 | 0.221 |
| 18:1w7 | gram - | -0.188 | -0.194 | -0.166 |  | 0.087 | -0.330 | 0.053 |
| cy19:0 | gram - | 0.040 | 0.264 | -0.098 |  | 0.111 | 0.119 | 0.261 |
| 10Me16:0 | actino | 0.036 | 0.202 | 0.058 |  | 0.149 | -0.053 | -0.314 |
| 15:0 | bacteria | 0.277 | -0.134 | -0.023 |  | -0.275 | 0.233 | 0.005 |
| 17:0 | bacteria | 0.086 | 0.079 | -0.351 |  | 0.087 | 0.309 | 0.171 |
| 17:1w6 | bacteria | 0.147 | 0.173 | 0.106 |  | -0.092 | 0.084 | 0.129 |
| 18:1w5 | bacteria | -0.245 | 0.012 | -0.239 |  | 0.254 | -0.174 | 0.050 |
| 16:1w5 | fungi | -0.002 | -0.376 | 0.081 |  | -0.187 | -0.311 | 0.098 |
| 18:1w9 | fungi | -0.327 | -0.131 | -0.056 |  | 0.213 | -0.232 | 0.102 |
| 18:2w6 | fungi | -0.311 | -0.173 | 0.054 |  | 0.081 | -0.285 | 0.265 |
| 18:3w3 | fungi | -0.230 | -0.088 | 0.132 |  | 0.023 | -0.222 | 0.057 |
| i14:0 | general | 0.315 | -0.029 | 0.107 |  | -0.295 | 0.000 | -0.164 |
| 14:0 | general | 0.296 | -0.123 | 0.205 |  | -0.308 | 0.054 | -0.128 |
| 16:0 | general | 0.119 | 0.180 | 0.052 |  | 0.042 | 0.358 | 0.195 |
| 16:1w11 | general | 0.101 | -0.322 | 0.015 |  | -0.215 | -0.271 | 0.108 |
| 18:0 | general | 0.029 | 0.240 | 0.182 |  | 0.111 | 0.043 | -0.186 |
| 19:1w8 | general | -0.255 | 0.065 | -0.214 |  | 0.276 | -0.087 | -0.174 |
| 20:0 | general | 0.049 | 0.297 | -0.281 |  | 0.273 | 0.182 | -0.083 |

Table S2. Factor loadings for the first three axes of the Principal Component Analyses used in structural equation models.

PCAs have been performed individually for regular soil, including organic topsoil (O), mineral topsoil (A) and mineral subsoil (B), and for cryoturbated material (J). Markers for individual groups are assigned as following: gram positive bacteria (gram +), gram negative bacteria (gram -), actinobacteria (actino), general bacterial markers (bacteria), fungal markers (fungi) and unspecific markers (general).
